# Risk of Bias Assessment of Randomized Controlled Trials: From the 1994 Cochrane Collaboration Toolkit to ROBUST‐RCT

**DOI:** 10.1111/jep.70328

**Published:** 2025-11-27

**Authors:** Rafael Leite Pacheco, Rachel Riera

**Affiliations:** ^1^ Centre of Health Technology Assessment Hospital Sírio‐Libanês Sao Paulo Brazil; ^2^ Disciplina de Economia e Gestão em Saúde Universidade Federal de São Paulo (Unifesp) Sao Paulo Brazil; ^3^ Departamento de Medicina Centro Universitário São Camilo Sao Paulo Brazil; ^4^ Escola Paulista de Medicina Universidade Federal de São Paulo (Unifesp) Sao Paulo Brazil

## 1 Earlier Recommendations by the Cochrane Collaboration

In March 1994, the Cochrane Collaboration issued its inaugural formal guidance on the methodology for conducting and updating systematic reviews, presented as a section within the Cochrane Collaboration toolkit. This section, titled ‘Section VI: Preparing and Maintaining Systematic Reviews’, offered directives for assessing the methodological quality of randomized controlled trials incorporated into the reviews. It drew upon a prior study by Moher et al. [1], which systematically appraised scales and checklists devised to evaluate trial quality, including the Jadad scale [2]. The authors concluded that none of the existing scales at that time could be recommended without concerns.

The newly proposed framework for assessing methodological quality rested on four bias domains, termed ‘categories’ within the document: selection, performance, exclusion, and detection. The selective reporting of significant results was also noted as a criterion that might prove pertinent in certain contexts, though it was not considered as a fundamental domain.

The evaluation system delineated three possible ratings for the overall risk of bias: low, moderate, or high. Review authors were tasked with determining whether the trials fulfilled valid criteria across all domains (signifying a trial with a low risk of bias), whether some criteria were only partially satisfied (indicating a moderate risk), or whether one or more criteria remained unmet (denoting a high risk of bias).

A noteworthy excerpt from the document declares:

More complex scoring systems take more time to complete. They have not been shown to provide more reliable assessments of quality. They carry a greater risk of confusing the quality of reporting with the quality of the trial.

This underscores that the necessity of striking a balance between complexity and methodological rigour was already a matter of concern.

## 2 The Cochrane Collaboration Handbook (Versions 3 and 4)

In October 1996, Section VI was revised and reissued as a standalone publication, henceforth titled the Cochrane Collaboration Handbook. This third edition emphasizes the importance of critically appraising studies, shifting away from the earlier terminology of methodological quality assessment.

The principal recommendation retained the same four bias domains: selection, performance, attrition, and detection. The domain formerly designated as exclusion bias was renamed as attrition bias. The evaluation framework preserved its established logic for classifying the overall risk of bias as low, moderate, or high.

In 1999, the Cochrane Handbook was refined into its fourth version, preserving the recommendations for trial assessment with no substantial alterations. The fourth version of the handbook had some minor updates, including changes to its name in different versions. For example, version 4.2.1 [3] was named ‘Cochrane Reviwers' Handbook’ while version 4.2.6 [4] was named ‘Cochrane Handbook for Systematic Reviews of Interventions’, a designation that endures in the present version.

## 3 The Cochrane Handbook for Systematic Reviews of Interventions (Version 5) and the Cochrane Risk of Bias Tool

In the 11th volume of the Cochrane Collaboration Methods Groups Newsletter, released in June 2007, Julian Higgins and Douglas Altman foreshadowed the principal attributes of an innovative tool then under development from 2005 to 2007, designed expressly for evaluating the risk of bias in randomized controlled trials. Through this novel instrument, the authors established that the risk of bias constitutes a distinct concept from methodological quality.

The new tool was designed to encompass six domains: sequence generation, allocation concealment, blinding of participants, personnel, and outcomes, incomplete outcome data, selective outcome reporting, and other sources of bias.

The evaluation system would feature a domain‐specific judgement categorized as high, low, or unknown risk. The intermediate classification of moderate was discontinued, with a formal designation of unknown risk introduced in its stead.

A further significant advancement in this document lies in the recognition that certain domains would pertain to the assessment of individual outcomes rather than a broader study‐level evaluation, as elucidated in the subsequent passage:

Some sources of bias, such as blinding and incomplete outcome data, may differ for different outcomes, so authors will be encouraged to assess them separately for different outcomes.

This tool also emphatically clarified the necessity for transparency in each distinct judgement.

For each domain, the tool will require authors to provide a description of what happened, using verbatim quotes where appropriate.

In February 2008, the Cochrane Handbook was updated to its fifth version [5]. This new version incorporated the tool, then referred to as the ‘Cochrane Risk of Bias Tool.’ One terminological change was adopted from the preliminary version and the term ‘unknown risk of bias’ was replaced with ‘unclear risk of bias.’

This version of the Cochrane Risk of Bias Tool featured straightforward questions that could be answered with a simple ‘yes’ or ‘no’ to assess the risk of bias in each domain. For example, the first domain was assessed with the question ‘Adequate sequence generation?’, which would support a ‘yes’ or ‘no’ response.

In March 2011, the Cochrane Handbook was updated to version 5.1 [6]. In this version, the domain ‘blinding of participants/personnel/outcomes’ was split into two domains: ‘blinding of participants and personnel’ (performance bias) and ‘blinding of outcome assessment’ (detection bias).

Another notable difference was the removal of questions previously used to assist in making judgements:

The judgements are now expressed simply as ‘Low risk’, ‘High risk’ or ‘Unclear risk’ of bias. The questions have been removed, along with the responses ‘Yes’ indicating low risk of bias and ‘No’ indicating high risk of bias.

The Cochrane Collaboration's Risk of Bias Tool was formally published in *The BMJ* in 2011 [7]. The manuscript also proposed a method for formulating a summary assessment of risk of bias for each outcome within a trial, establishing an overall assessment for each outcome.

The overall assessment would be expressed as: (i) ‘low risk of bias for all key domains’, meaning that ‘bias, if present, is unlikely to seriously affect the results’; (ii) ‘low or unclear risk of bias for all key domain’, meaning that ‘there is a risk of bias that raises some doubt about the results’; and (iii) ‘bias may seriously affect the results’, meaning that ‘here is a high risk of bias in one or more key domains’.

Following its publication, several meta‐research studies identified issues with the use of the tool [8]. These studies analyzed samples of Cochrane reviews and found poor inter‐reviewer agreement on judgements [8], inconsistent approaches to reaching overall judgements [9], and inadequate application across all domains of the tool [10, 11, 12, 13, 14]. Similar problems were also observed in non‐Cochrane systematic reviews [15].

## 4 The Cochrane Handbook for Systematic Reviews of Interventions (Version 6) and the Cochrane Risk of Bias Tool 2.0

In July 2019, the sixth version of the Handbook introduced an updated risk of bias tool known as the Cochrane Risk of Bias Tool 2.0 (RoB 2.0), which included significant changes compared to its original version [16].

RoB 2.0 consists of five domains: (i) bias arising from the randomization process, (ii) bias due to deviations from intended interventions, (iii) bias due to missing outcome data, (iv) bias in measurement of outcomes, and (v) bias in selection of the reported result. The domain ‘other bias’ was removed.

While the domains are related, they do not mirror the original tool exactly. For instance, the domain ‘bias in selection of the reported result’ does not address bias due to selective non‐reporting of results, which was considered in the domain ‘selective outcome reporting’ in the original tool. Another example is the inclusion of considerations of baseline differences in the domain ‘bias arising from the randomization process,’ which was typically addressed in the ‘other bias’ domain in the original version.

RoB 2.0 also reintroduced two noteworthy features from the past. The first is an intermediate judgement, previously referred to as ‘moderate risk’ and now termed ‘some concerns.’ As a result, each domain can now receive one of three judgements: high risk of bias, some concerns, or low risk of bias.

The second feature involves the reintroduction of signalling questions. Each domain is assessed within a framework of signalling questions that can be answered as yes, probably yes, probably no, no, or no information.

The new version also distinguishes the risk of bias based on the nature of the effect of interest, specifically differentiating between the effects of assignment to the intervention and adherence to it. Judgements may vary due to the differing nature of these effects.

RoB 2.0 provides a clear framework for assessing the overall risk of bias for each evaluated outcome. It also offers complex algorithms for the potential responses to the signalling questions, which inform the judgements for each domain.

## 5 Challenges in RoB 2.0 Implementation

RoB 2.0 encountered challenges in its implementation shortly after its release. The authors of this commentary participated in a RoB 2.0 workshop at the 25th Cochrane Colloquium in Edinburgh, where the audience was unable to reach a consensus on how to approach the first domain, even after 2 h of intense discussion.

Early investigations revealed low adherence to the tool in both Cochrane [17] and non‐Cochrane reviews [18], as well as poor inter‐rater reliability [19] and inadequate usage [18]. Many attributed the slow pace of implementation to the significant increase in the complexity of the assessment process [20].

## 6 The ROBUST‐RCT (Risk of Bias Instrument for Use in Systematic Reviews‐For Randomized Controlled Trials)

In March 2025, the authors affiliated with GRADE Working Group published a new tool called ROBUST‐RCT [21], designed to assess the risk of bias in randomized controlled trials. The primary rationale for developing a new tool was the call for simplicity and practicability, which had been sacrificed in favour of methodological rigour in previous tools.

ROBUST‐RCT consists of six core domains: random sequence generation, allocation concealment, blinding of participants, blinding of healthcare providers, blinding of outcome assessors, and outcome data not included in the analysis. The judgement for each domain has four possible outcomes: low, probably low, probably high, or definitely high.

For most domains, the process to reach each judgement is very similar to the earlier version of the Cochrane risk of bias tool from 2008. Judgements are made by answering straightforward questions, such as ‘Was the allocation sequence adequately generated?’ and ‘Was the allocation adequately concealed?’

ROBUST‐RCT also includes optional domains that reviewers may choose to incorporate if deemed relevant. One of these optional domains pertains to selective outcome reporting, while other items partially overlap with assessments included in the ‘other bias’ domain from the first version of the Cochrane Collaboration tool.

## 7 The Long Road Ahead

We believe that ROBUST‐RCT will be more swiftly accepted by the scientific community as it addresses the issues that led to the high complexity of RoB 2. However, we feel that ROBUST‐RCT shares many characteristics with the original version of the Cochrane risk of bias tool (Table 1).

**Table 1:** Domains and key features of different versions of the Cochrane risk of bias tool and ROBUST‐RCT.

Version/Tool	Year	Domains	Judgement categories	Key features/Notes
Cochrane Collaboration toolkit. Section VI: Preparing and Maintaining Systematic Reviews	1994	Selection	Low	First formal guidance from Cochrane.
		Performance bias	Moderate	Emphasized simplicity over complex scoring systems (based on previous work by Moher et al.).
		Exclusion bias	High	
		Detection bias		
		Selective reporting noted, but not a fundamental domain		
Cochrane Collaboration Handbook version 3.0	1996	Selection bias	Low	Emphasizes the importance of critically appraising studies, shifting away from the earlier terminology of methodological quality assessment.
		Performance bias	Moderate	Retained earlier framework with minor terminology changes.
		Attrition (renamed from Exclusion)	High	
		Detection bias		
Cochrane Handbook for Systematic Reviews of Interventions version 4.2.6	1999	Same as 1996	Same as 1996	Core structure retained.
				No major methodological changes.
Cochrane risk of bias tool (Cochrane Collaboration Methods Groups Newsletter)	2007	Sequence generation	Low	Established that the risk of bias constitutes a distinct concept from methodological quality.
		Allocation concealment	High	Reiterates outcome level assessment for some domains (blinding and incomplete outcome data).
		Blinding of participants, personnel, and outcomes	Unknown	Emphatically clarified the necessity for transparency in each distinct judgement.
		Incomplete outcome data		
		Selective outcome reporting		
		Other sources of bias		
				
Cochrane risk of bias tool (Cochrane Handbook for Systematic Reviews of Interventions version 5.0)	2008	Sequence generation	Low	‘Unknown risk of bias’ was replaced with ‘unclear risk of bias’.
		Allocation concealment	High	Introduced straightforward questions that could be answered with a simple ‘yes’ or ‘no’ to assess the risk of bias in each domain.
		Blinding of participants, personnel, and outcomes	Unclear	
		Incomplete outcome data		
		Selective outcome reporting		
		Other sources of bias		
Cochrane risk of bias tool (Cochrane Handbook for Systematic Reviews of Interventions version 5.1)	2011	Sequence generation	Low	The domain ‘blinding of participants/personnel/outcomes’ was split into two domains.
		Allocation concealment	High	Removal of questions previously used to assist in making judgements.
		Blinding of participants and personnel	Unclear	
		Blinding of outcome assessment		
		Incomplete outcome data		
		Selective outcome reporting		
		Other sources of bias		
Cochrane risk of bias tool 2.0 (Cochrane Handbook for Systematic Reviews of Interventions version 6.0)	2019	Bias arising from the randomisation process.	Low	Return of an intermediate judgement, some concerns (previously referred as moderate risk of bias).
		Bias due to deviations from intended interventions.	Some concerns	Removed other bias domain.
		Bias due to missing outcome data.	High	Distinguishes the risk of bias based on the nature of the effect of interest.
		Bias in measurement of outcomes.		Reintroduction of signalling questions that can be answered as yes, probably yes, probably no, no, or no information
		Bias in selection of the reported result.		Introduces algorithms for the potential responses to the signalling questions, which inform the judgements for each domain.
ROBUST‐RCT	2025	Random sequence generation, Allocation concealment	Low	Calls for simplicity and practicability.
		Blinding of participants	Probably low	
		Blinding of healthcare providers	Probably high	Judgements are made by answering straightforward questions, similarly to the 2008 version of the Cochrane risk of bias tool.
		Blinding of outcome assessors	Definitely high	Contains optional domains, which include selective outcome reporting and other items partially overlap with assessments included in the ‘other bias’ domain from the first version of the Cochrane risk of bias tool.
		Outcome data not included in the analysis.		

RoB 2 was developed to overcome the limitations identified by years of meta‐research, which highlighted inadequate usage and low replication of judgements in the original Cochrane risk of bias tool.

Will the decision to prioritize simplicity reintroduce past problems? Future research will provide the answer to this question.

Several other tools and different frameworks for assessing randomized controlled trials were also proposed in the abovementioned period, with special mention to JBI Critical appraisal checklist of randomized controlled trials [22]. This commentary does not aim to cover the entire debate surrounding all proposed tools; rather, it seeks to provide a historical perspective on the methodological development of the Cochrane approach for assessing randomized controlled trials. Additionally, it compares certain features of the ROBUST‐RCT with previous versions of the Cochrane Risk of Bias tool.

In recent years, methodological advances have led to an accumulation of different and sometimes redundant tools designed to aid the conduct and reporting of systematic reviews. This commentary summarizes nearly 30 years of the development of some proposed tools for assessing the risk of bias of randomized controlled trials. The future requires more attention to how we implement these tools, not just to how we develop them.

## Conflicts of Interest

The authors declare no conflicts of interest.
